# Novel integrated workflow allows production and in-depth quality assessment of multifactorial reprogrammed skeletal muscle cells from human stem cells

**DOI:** 10.1007/s00018-022-04264-8

**Published:** 2022-04-09

**Authors:** Dinis Faustino, Heinrich Brinkmeier, Stella Logotheti, Anika Jonitz-Heincke, Hande Yilmaz, Isil Takan, Kirsten Peters, Rainer Bader, Hermann Lang, Athanasia Pavlopoulou, Brigitte M. Pützer, Alf Spitschak

**Affiliations:** 1 Institute of Experimental Gene Therapy and Cancer Research, Rostock University Medical Center, 18057 Rostock, Germany; 2 Department Life, Light and Matter, University of Rostock, 18059 Rostock, Germany; 3 Institute of Pathophysiology, University Medicine Greifswald, 17489 Greifswald, Germany; 4 Biomechanics and Implant Technology Research Laboratory, Department of Orthopedics, Rostock University Medical Centre, 18057 Rostock, Germany; 5 Izmir Biomedicine and Genome Center (IBG), Balcova, 35340 Izmir, Turkey; 6 Izmir International Biomedicine and Genome Institute, Dokuz Eylül University, Balcova, 35340 Izmir, Turkey; 7 Department of Cell Biology, Rostock University Medical Center, 18057 Rostock, Germany; 8 Department of Operative Dentistry and Periodontology, Rostock University Medical Centre, 18057 Rostock, Germany

**Keywords:** Multipotent stem cells, Myogenic differentiation, Adenovector-mediated MYOD expression, Small molecules, Electrical pulse stimulation, Skeletal muscle transcriptomics, Myo-informatics

## Abstract

Skeletal muscle tissue engineering aims at generating biological substitutes that restore, maintain or improve normal muscle function; however, the quality of cells produced by current protocols remains insufficient. Here, we developed a multifactor-based protocol that combines adenovector (AdV)-mediated MYOD expression, small molecule inhibitor and growth factor treatment, and electrical pulse stimulation (EPS) to efficiently reprogram different types of human-derived multipotent stem cells into physiologically functional skeletal muscle cells (SMCs). The protocol was complemented through a novel in silico workflow that allows for in-depth estimation and potentially optimization of the quality of generated muscle tissue, based on the transcriptomes of transdifferentiated cells. We additionally patch-clamped phenotypic SMCs to associate their bioelectrical characteristics with their transcriptome reprogramming. Overall, we set up a comprehensive and dynamic approach at the nexus of viral vector-based technology, bioinformatics, and electrophysiology that facilitates production of high-quality skeletal muscle cells and can guide iterative cycles to improve myo-differentiation protocols.

**Supplementary Information:**

The online version contains supplementary material available at 10.1007/s00018-022-04264-8.

## Introduction

Engineered skeletal muscle holds great potential for disease modeling, drug discovery, and regenerative medicine. Critical to this effort is the development of tools that enable natural autologous therapeutic strategies, usable to overcome current barriers for treating muscular impairments due to traumatic injuries or neuromuscular diseases that lack long-term treatments to fully restore normal tissue function. Skeletal muscle is the most abundant tissue type in the adult human body and its maintenance is important not only for locomotion but also for metabolic homeostasis. It entails innervated, voluntary muscle cells that exhibit contractility, excitability, extensibility, and elasticity based on its distinctive tissue organization. Skeletal muscles are composed of muscle fiber bundles formed by the fusion of undifferentiated myoblasts into long cylindrical, multinucleated structures called myotubes. While early myotubes are characterized by gene expression of embryonic and postnatal isoforms of muscle-specific genes, innervated mature muscle fibers express the adult isoforms of slow-twitch or fast-twitch skeletal muscle. Within myofibers, actin and myosin generate a contractile machinery of repeating units of sarcomeres that are responsible for the typical striated appearance and functions of the muscles [1]. The muscle fibers are classified according to their myosin heavy chain (MYHC) isoforms, with each type serving different metabolic energy requirements and locomotion patterns: type I fibers contain myoglobin, rely on oxidative phosphorylation, express slow-twitch MYHCs and are suitable for endurance. Type II fibers lack myoglobin, rely on glycolysis, express fast-twitch MYHCs and are suited for fast bursts of power [1].

Mature skeletal muscles show remarkable regenerative potential that is normally maintained by satellite cells (SCs), a reservoir of muscle-specific precursor cells located along muscle fibers, responsible for postnatal muscle growth and regeneration of injured tissue via microenvironmental interplay [2]. However, in several pathological conditions, this natural regeneration capacity is either impaired or insufficient to replenish the tissue mass. This necessitates artificial regeneration of muscle fibers that are used as implants in reconstructive muscle surgery. Skeletal muscle tissue engineering (SMTE) aims at the development of artificial tissues capable of repairing or replacing normal function of defective muscles [3]. In this respect, cells harvested either from the patient or a donor are cultured in vitro, with or without the use of tissue scaffolds, to generate functional muscle tissue that can be implanted in the patient's body. The produced cells should ideally mimic skeletal muscle tissue not only in composition but also in functionality and contractibility [1].

A widely used approach in SMTE is the reprogramming of several cellular sources to muscle tissue via exploitation of major transcription factors (TF) that regulate the myogenic lineage. In particular, during muscle development, precursor cells are activated to satellite cells, which are further proliferated to myoblasts that, in turn, differentiate to myocytes. Finally, myocytes are fused with either one another and/or residual myofibers and regenerate muscle tissue. This process is controlled by a hierarchy of transcription factors which act in a spatiotemporal-dependent manner [4]. Re-expression of master regulators of myo-differentiation in pluripotent cells is an established STME strategy, while MYOD, which catalyzes transformation of the myoblasts to myocytes has been primarily recruited in TF-based reprogramming protocols.

Such approaches nevertheless pose challenges on multiple levels that slow their overall translation into transplantation therapies. First, MYOD-induced transdifferentiation remains essentially incomplete, which is associated with inadequacies in chromatin remodeling [5]. Although MYOD is a key for myoblast-to-myocyte differentiation, it acts to inhibit proliferation and therefore cannot maintain the proliferative and regenerative potential of normal skeletal muscle. The inherently complex patterns of TF-mediated regulation of natural myo-differentiation also suggest that a single TF might be inadequate for efficient and complete skeletal muscle reprogramming. Instead, hierarchical and spatiotemporally-dependent programs of myo-differentiation are theoretically more compatible with multistep, multifactorial protocols of SMTE. In this case, combinatorial induction and/or silencing of key TFs at appropriate time points in conjunction with timed environmental stimuli may have the potential to more faithfully recapitulate the natural myogenic process and to maximize the output of engineered cells while avoiding the generation of heterogeneous cell populations. Another important aspect of SMTE is the question of which cells can be efficiently reprogrammed into skeletal muscle. Although use of autologous muscle satellite cells appears to be a viable therapeutic strategy, this approach has limited success due to the lower regenerative capacity of cultured compared with freshly isolated cells, poor survivability and migration of transplanted cells, adverse immune responses, and inability to fuse with injured host myofibers [6]. Hence, there is a need for alternative cell sources that are accessible, abundant, and suitable for skeletal muscle reprogramming. Searching for multipotent stem cells (MSCs) with high proliferative and myogenic potential, various mesenchymal cells have been investigated [1, 6]. In this respect, dental stem cells are becoming extremely relevant for tissue engineering and regenerative medicine due to their remarkable self-renewal capacity and multi-differentiation potential [7], although their ability to produce muscle cells is unknown.

Establishing multifactorial reprogramming protocols that mimic a dynamic myogenic process in a purely experimental way essentially necessitates tedious testing of a plethora of factors at many time points in different cellular contexts. Computational methods can significantly accelerate these efforts by (a) assessing the function and maturity of the reprogrammed tissue and (b) retroactively predicting molecular factors that can be further modulated toward full skeletal muscle reprogramming. Herein, we developed a programming protocol that is based on an optimized two-step differentiation process driven by a three-method multifactor combination approach consisting of AdV-mediated MYOD expression, small molecule inhibitors, FGF-2, and electrical pulse stimulus. In this pilot study, we used human dental follicular stem cells (hDFSC) in addition to adipose tissue-derived multipotent mesenchymal stem cells (hADMSC), and dermal fibroblasts (hFB) as starting material. Complementing this protocol, we developed a novel in silico workflow to estimate the quality and functionality of generated muscle tissue. Overall, we provide a comprehensive pipeline at the interface of SMTE and bioinformatics that can facilitate the production of high-quality skeletal muscle cells (SMC) and guide further iterative cycles to improve the protocol.

## Materials and methods

### Adenovector production

For generation of the adenoviral (Ad) vector Ad.MyoD, the plasmid LV-TRE-WT human MyoD-T2A-dsRedExpress2 (Addgene #60,628) was digested with BamHI and EcoRV. The resulting MyoD-T2A-dsRedExpress2 cassette was treated with DNA-Polymerase I, Large (Klenow) Fragment (NEB) and subsequently inserted into the EcoRV site of the pShuttle vector (Agilent, Santa Clara). After sequencing, pShuttle MyoD-T2A-dsRedExpress was used to generate Ad vector in 293 cells as described [8]. Infection was carried out in all three cell types at a multiplicity of infection (MOI) of 100 that allows 100% transduction of cultured cells.

### Cell culture

Human ADMSC were isolated from lipoaspirate samples of healthy individuals who underwent liposuction or lipofilling. The detailed process of hADMSC isolation and characterization has been described previously [9]. Dental follicle stem cells were isolated from dental follicles of extracted wisdom teeth before tooth eruption as described earlier [10]. Briefly, after removal of the tooth, the follicle was removed and subjected to enzymatic treatment. Subsequently, cells were seeded on tissue flasks containing DMEM-F12 (Gibco) supplemented with 10% FCS and 1% Zellshield (Minerva Biolabs) and obtained by plastic adherence. Both mesenchymal stem cell types and human fibroblasts were maintained at 37 °C and 5% CO_2_ humidified atmosphere. Medium was changed every 2–3 days. Sub-cultivation was performed when cells reached a confluency of ~ 90%. All donors have given their written consent for the donation of their tissue according to the Declaration of Helsinki. The studies were approved by the ethical committee of the Medical Faculty of Rostock University (registration number: hADMSC: A 2014–0092, hDFSC: A 2017–0158).

### XTT-Assay

The XTT assay has been performed as previously described [11]. Briefly, 10 × 10^3^ cells were seeded on a 96-well plate (Corning Inc.). XTT working solution was freshly added every 24 h after Ad.MyoD transduction (MOI 100) in all three cell types and absorbance was measured at 490 nm over 3 days.

### Myogenic differentiation

Biolaminin (Biolamina) was applied to 35-mm dishes and the coated dishes were pre-incubated overnight at 4 °C. After seeding approximately 25 × 10^3^ cells per cm^2^, cells are incubated for 24 h in respective cell maintenance medium. Subsequently, the tissue was washed with PBS and supplemented with 1 ml of medium containing either Ad.MyoD. After 30 min incubation (37 °C, 5% CO_2_), adenoviral supernatant was removed and 3 ml of differentiation medium supplemented with 7.5 µM CHIR99021 (Tocris) and 0.5 µM LDN193189 (Tocris) added. After 72 h, cells were washed with PBS and the differentiation medium was renewed supplemented with 20 ng/µl FGF-2 (Tocris). Every second day until day 14 of the experiment, differentiation medium was refreshed, and from day 7 to 14 electric pulse stimulation (EPS) was applied to the cells. See Supplemental Information Table S1 for the differentiation medium composition.

### Electric pulse stimulation (EPS)

The experimental system is based on a commercially available six well cell culture plate (Corning Inc.). To generate capacitively coupled electric fields, two titanium electrodes (anodized with DOTIZE® by DOT GmbH, Rostock, Germany) were placed opposite to each other on the outside of the well. The three electrodes on each side were connected by the same conductive material as the electrodes. The electrodes were fitted as closely as possible to the polystyrene wells. A notch at the end of the electrodes ensured power supply via attaching a cable with a crocodile clip. A petri dish with fixed cell seeded scaffolds was placed in this system. To apply sinusoidal 0.3 voltage with a frequency of 1 kHz, a function generator (GX 310, Metrix, Annecy-le-Vieux, France) was used. A custom-made timer allowed automated stimulation for 45 min three times per day following the protocol of [12]. The stimulation system itself was placed in an incubator to provide a stable and hypoxic environment (37 °C, 5% CO_2_) for the cells.

### RNA isolation, RT-PCR and qPCR analysis

RNA isolation, reverse transcription, RT-PCR and qPCR were performed as described earlier [11]. RNA was extracted with NucleoSpin® miRNA kit (Macherey–Nagel). For qRT-PCR, cDNA was added to iTaq™ Universal SYBR Green Supermix (Bio-Rad) and analyzed using CFX96 Touch™ RealTime PCR Detection System (Bio-Rad). Relative gene expression was calculated by comparative CT method using RPLP0 for normalization. Statistical analyses were evaluated using Student’s *t* test, and *p* < 0.05 was considered statistically significant. Primer sequences used for PCR are indicated in Supplemental Information Table S2.

### Immunofluorescence

Cells grown on glass coverslips (Thermo Scientific) were fixed with −20 °C methanol (JT Baker) for 5 min, permeabilized for 2 h with 10% normal goat serum (Thermo Fisher) and 0.3% Triton X-100 in PBS. After treatment with blocking buffer (1% BSA in PBS/0.3% Triton X-100), immunofluorescence staining of the cells was performed by incubation with primary anti-sarcomeric α-actinin antibody (Abcam, ab68167) diluted in 1% BSA in PBS/0.3% Triton X-100, washing in PBS, and incubation for 1 h with Cy5-labeled secondary antibody (Invitrogen A10523). Images were obtained with an inverted confocal laser-scanning microscope (Zeiss).

### Patch clamp and electrophysiological recordings

Whole-cell Na + currents and K + currents were recorded at room temperature using an EPC 10 patch-clamp amplifier (HEKA Elektronik GmbH, Lambrecht, Germany). Pipettes were filled with standard internal solution or KCl solution and had resistances of 2–3 MΩ. The standard internal solution was composed of (in mM): 140 CsCl, 1.4 MgCl_2_, 10 EGTA, and 10 HEPES–CsOH; and the KCl solution of: 100 K gluconate, 20 KCl, 1 CaCl2, 1 MgCl_2_, 10 HEPES, 11 EGTA–KOH, 4 ATP-Mg^2+^, 3 phosphocreatine disodium salt x H_2_O and 9 sucrose. The external solution for all recordings including action potentials contained (in mM): 140 NaCl, 2.5 KCl, 2 CaCl_2_, 1.2 MgCl_2_, 5 CsCl 10 HEPES, 5 D(+) glucose, pH 7.4. To determine the voltage-dependence of activation of the Na + currents, a cyclic pulse program was applied, each cycle consisting of a constant 100-ms prepulse to −135 mV and a 10-ms test pulse, varied between −80 mV to + 40 mV in 5-mV steps. The peak currents were plotted against the test potential (current/voltage curves). The voltage dependence of steady-state activation of the Na + channels was derived from these plots. Finally, Boltzmann equations were fitted to the data points. To study the voltage dependence of steady-state inactivation of the Na + channels, another cyclic pulse program was applied with each cycle consisting of a 100-ms prepulse that was varied between −120 mV and −10 mV in 5-mV steps followed by test pulses to −10 mV (for 10 ms). The obtained current peaks were normalized and plotted against the prepulse potential. Finally, Boltzmann curves were fitted to the data points [13]. To test for the presence of K + outward currents of the myotubes, we applied the same pulse protocol as described above for the current–voltage curves of the Na + currents, but prolonged the test pulses to 50 ms. The KCl containing internal solution was used for recording of the K + currents. Amplitudes of K + outward currents were evaluated from the last 20 ms of the traces and used for I/V curves. After finishing the recordings in the voltage-clamp mode and only if using the internal KCl solution, the amplifier was switched to current clamp mode. Then, depolarizing currents of 100-ms duration were injected to test for cellular excitability, i.e., the ability of the cells to generate action potentials (APs). For most of the myotubes between few pA and 100 pA were required to elicit APs. For each cell, the minimum current injection, sufficient for the induction of APs was determined. This information was then used to induce trains of 10 APs at a frequency of 1 Hz.

### In silico reconstruction of the gene regulatory network underlying normal skeletal muscle phenotype and function.

For the reconstruction of the gene regulatory network (GRN), the following steps were performed: (a) Acquisition of RNA-seq data from normal skeletal tissue samples: Non-normalized, raw RNA-Seq read counts of muscle skeletal normal tissue samples were downloaded from the Genotype-Tissue Expression (GTEx) Portal (https://gtexportal.org/home/) using the ‘TCGAbiolinks’ package. Collectively, 475 normal muscle skeletal GTEx samples were downloaded through the Recount2 project using the function TCGAquery_recount2 in the ‘TCGAbiolinks’ package, as Ranged Summarized Experiment (RSE) objects. The raw counts were scaled with the ‘scale_counts’ function in the ‘Recount’ package (v.1.16.1) [14, 15]. The RNA consensus tissue gene data from the Human Protein Atlas (HPA) [16], which contain the transcriptomes of a large sample of mature normal skeletal muscle tissues, were also downloaded. For the analysis, we extracted the GTEx samples and included the genes that are common between GTEx and HPA skeletal muscle datasets.

(b) Weighted gene co-expression network analysis: Considering that genes with similar expression patterns are usually implicated in similar biological processes [17], we screened for the co-expressed transcripts among all the genes in the transcriptomes of skeletal muscle tissue samples. To this end, a co-expression network was constructed by Weighted Gene Co-expression Network Analysis of Muscle-Skeletal Normal Tissues from GTEx [18], so as to identify modules (i.e., clusters of densely connected co-expressed genes) with similar expression patterns and exclude outliers. After exclusion of outliers, the gene co-expression network was reconstructed based on the 473 muscle samples using the package WGCNA (v.1.69) [18] implemented in R. The values of the genes were converted to FPKM values using the function count2FPKM in the RNAAgeCalc package. Analysis was conducted using the log2 (fpkm + 1) value. The goodSamplesGenes function was used to iteratively filter genes and samples. The soft-threshold power was applied to construct pairwise Pearson’s correlation matrices so as to measure gene co-expression similarities. The function pickSoftThreshold was used to analyze network topology and select a suitable soft-thresholding power. The soft-thresholding power (*β*) of 14 (scale-free *R*^2^ = 0.8) was chosen by applying the approximate scale-free network topology criterion. The adjacency matrices were calculated with the function adjacency, which converts the Pearson’s correlation coefficients into gene connection strengths. The adjacency matrices were transformed into Topological Overlap Matrix (TOM) with the function TOMsimilarity to minimize the effects of noise. To group genes into modules, the average linkage hierarchical clustering method was applied using the function hclust to cluster module eigengenes (i.e., first principal component) with TOM-based dissimilarity measure, by setting the cut height at 0.25 for module merging and the β value at 14. A relatively high minimum module size of thirty (minModuleSize = 30) was selected. The dynamicTreeCut algorithm was applied to detect modules with eigengenes correlation coefficient above 0.75, by setting the cut height threshold (MEDissThress) at 0.25. The WGCNA dendrogram is shown in Supplemental Fig. S2.

(c) GO term-based identification of human protein coding genes involved in skeletal muscle development and function: To identify genes that are important for skeletal muscle development and function, we used relevant keywords to mine for GO terms in Mouse Genome Informatics database, such as skeletal muscle, Z disk, I band, M band, striated muscle, sarcomeres, muscle fiber, slow- and fast-twitch muscle fiber, troponin, tropomyosin, transition between fast and slow fiber, actinin, myoblast fusion, actin, etc. To this end, we downloaded the ‘GO terms and GO IDs for all three ontologies (Biological Process, Cellular Component, Molecular Function) (tab-delimited)’ files from MGI and filtered it using terms specific for skeletal muscle development and function, to retrieve a list of GO term IDs relevant for skeletal muscle function (GO ID). The list of the GO IDs was used as a query in the field “Gene Ontology (GO) classifications” of “Genes and markers query”, after selecting ‘protein coding gene’ at the field ‘Feature type’. We used the resulting gene list as input in the ‘Batch query’ option, selecting ‘Gene Ontology (GO)’ as output, to match each gene with the corresponding skeletal muscle function-related GO IDs and term descriptions. Using the abovementioned procedure, we generated a list with 1289 genes that are involved in GO terms characteristic for skeletal muscle phenotype and function (Supplemental Table S3). We identified approved human orthologues for the 1262 genes of them by HUGO database (Eyre et al. 2006) and reciprocal BLASTp, and the official HGNC gene symbols were used (Supplemental Table S4). Of those, 1023 were found to be also co-expressed in the transcriptome of the human skeletal muscle tissues.

(d) Functional Interaction Network analysis: The genes/gene products that are expressed in the skeletal muscle tissue and are important for its function are further interconnected, generating a regulatory network, whereby the most highly connected nodes, the so-called “hubs”, are considered biologically significant and more relevant to the overall function of the network [19–21]. The intra-modular hubs are central to a given network module, with the highest number of connections to the neighboring nodes, whereas inter-modular hubs are intermediate between two or more modules. To this end, the 1023 genes that were found co-expressed in human skeletal muscle and are involved in muscle function, were provided as input to the Search Tool for the Retrieval of Interacting Genes (STRING) (v.11.0) (https://string-db.org/) database [22] to investigate the functional and/or physical associations among their corresponding gene products. The highest confidence score ≥ 0.9 was set as a cutoff. We downloaded the matrix with pairwise associations and selected the hubs with more than 2 connections to the immediate neighboring node. For further specification of hub genes Cytoscape software (v.3.8.2) with the Cytoscape plugin CytoHubba for ranking nodes was utilized selecting hub genes that are associated with fiber type I and II specific marker genes [23], which are indicative of skeletal muscle cell maturation. Ranking was performed according to several topological analysis methods including Degree, Edge Percolated Component (EPC), Maximum Neighborhood Component (MNC), Density of Maximum Neighborhood Component (DMNC), Maximal Clique Centrality (MCC) and seven centralities (Bottleneck, EcCentricity, Closeness, Radiality, Betweenness, and Stress, Clustering Coefficient). Overall, we reconstructed a network underlying skeletal muscle phenotype and function which consists of 116 hubs (Supplemental Table S5). All analyses were performed in the R statistical computing environment v.4.0.3. (https://www.r-project.org). The heatmaps were generated using DESeq2.

### Identification of a muscle-restricted gene signature in normal skeletal muscle transcriptomes

We used the RNA consensus tissue gene data from the Human Protein Atlas, where consensus transcript expression levels are summarized per gene in 62 tissues based on transcriptomics data from three sources: HPA, GTEx and FANTOM5. The consensus normalized expression ("NX") value is calculated as the maximum NX value for each gene in the three data sources. We searched for genes with profound expression values NX > 1 in muscle tissues and, of those, we excluded the genes that are expressed in muscles plus at least one of any other site. This approach led to a muscle-restricted signature which comprises of 8 genes, namely DUPD1, KLHL38, METTL11B, MYH6, MYL7, OR5H2, OR6C70 and OR9Q1.

### Identification of stage-specific indicators of maintenance of the skeletal muscle cell phenotype via meta-analysis of RNA-seq myodifferentiation data

The RNAseq raw values of transcriptomes of cells from distinct stages of myodifferentiation [24] were downloaded from NCBI (GSE129505). For RNA-Seq transcriptome analysis, a pipeline was employed consisting of raw RNA-Seq read trimming with Trimmomatic [25], filtering rRNA with SortMeRNA, read quality control with FastQC [A Quality Control Tool for High Throughput Sequence Data [Online]. Available online at: http://www.bioinformatics.babraham.ac.uk/projects/fastqc/ (2015), "FastQC," https://qubeshub.org/resources/fastqc], alignment of reads to the human reference genome GRCh38 (Ensembl version 102) with the splice junction aligner STAR [26], read count quantification with HTSeq [27] and calculation of FPKM values PCGs for each stage of differentiation. The genes which start being expressed from each stage of myodifferentiation and on, as well as the 71 genes with patterns of increasing expression toward fully differentiated stages are included in Supplemental Table S6.

### Microarrays and gene set enrichment analysis

RNA from triplicates of fully differentiated skeletal muscle cells of reprogrammed hFBs, hADMSCs and hDFSCs (day 14) and undifferentiated MSCs/fibroblast controls was isolated and equal amounts of RNA were applied to Clariom™ D Assay (Thermo Fisher). Background-corrected signal intensities were determined, processed and normalized using the Transcriptome Analysis Console (TAC, Affymetrix) and the SST-RMA algorithm. Significantly differentially regulated targets (*p* value < 0.05, |∆|≥ twofold) in test samples versus corresponding controls were determined, as previously described [17]. For the Gene Set Enrichment Analysis, the GSEA-P 2.0 software (Broad Institute, Cambridge, Massachusetts) [28] was used. Enriched hallmark and Gene Ontology terms were plotted against the negative log10 of their individual FDR value (< 0.05).

## Results

### Establishment of a multifactorial-guided protocol for differentiation of human fibroblasts, adipose- and dental follicle-derived MSC toward the myogenic lineage

**Fig. 1:**
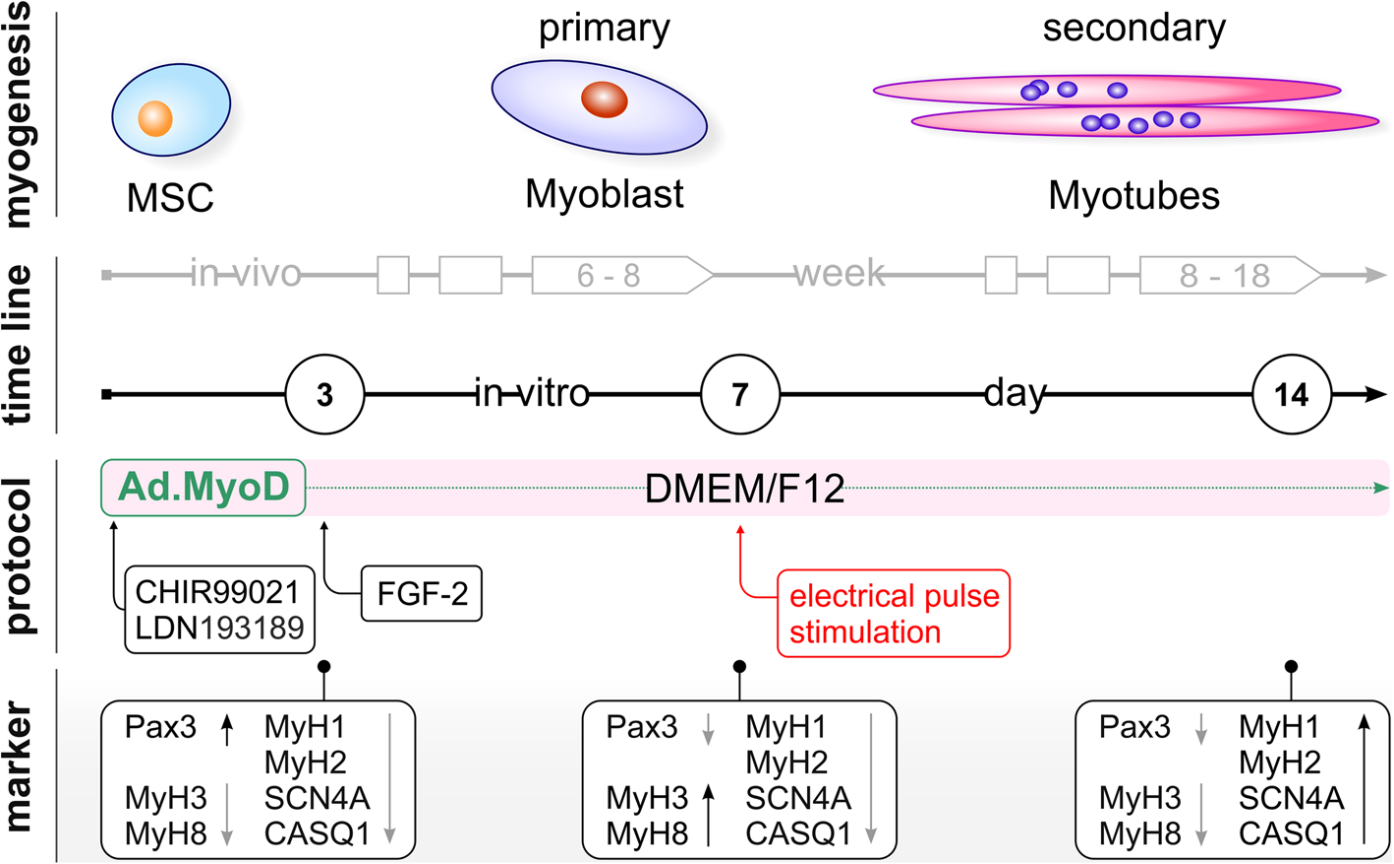
Overview of myogenic development steps in vivo and in vitro in combination with the applied multifactorial-based reprogramming protocol. Depicted are addtion of the MYOD-expressing Ad vector complemented with culture supplements and electrical pulse stimulation at distinct time points. Expression levels of marker genes representing characteristical myogenic developmental stages were quantified in reprogrammed cells to monitor the transdifferentiation progress

Transcription factors play a crucial role in cell differentiation and, together with small molecules and growth factors, can guide the activity of cells into specific phenotypes. To obtain fully reprogrammed skeletal muscle cells, we developed a straight forward three-method combination protocol (Fig. 1). For effective induction of myogenesis, we first generated a recombinant AdV encoding a MYOD red fluorescence (RFP) fusion protein (Ad.MyoD), which allows visualization of MYOD expression in transduced cells. To enhance the transdifferentiation process, overexpression of MYOD was supplemented by the addition of CHIR99021 (GSK3 inhibitor), LDN193189 (inhibitor of BMP type I receptors), and fibroblast growth factor 2 (FGF-2), which support paraxial mesoderm and counteract lateral mesoderm differentiation, directing development into skeletal muscle cells. Myogenesis is an evolutionarily conserved process comprising in vivo of two early developmental stages. The initial population of proliferating progenitor cells is characterized by high expression of paired-box transcription factors such as PAX3, which are downregulated with the onset of primary myogenesis between the sixth and eighth weeks of development, and primary myofibers are formed. In secondary myogenesis, approximately lasting from week eight to eighteen, secondary myofibers arise from fetal myoblasts. During these two phases, several isoforms of striated muscle myosin heavy chain (MYHC) are expressed, which convert chemical energy into mechanical force for muscle contraction. They are encoded by different genes that are regulated in a tissue- and development-specific manner [29].

Importantly, early fetal muscles predominantly express embryonic and perinatal MYHCs encoded by *MYH3* and *MYH8*, whereas later developmental stages (myotubes) are characterized by a switch to fast myosins *(MYH2* and *MYH1*). Mechanistically, the contractile apparatus is intertwined with ionic exchange at membranes, which is mediated by associated proteins such as the muscular sodium (Na^+^) channel Na_v_1.4 (*SCN4A*), responsible for action potential processing, and calsequestrin (*CASQ1*), a calcium binding protein in the sarcoplasmic reticulum. Thus, expression of these genes provides indispensable indicators for accurate assessment of myogenesis. To monitor the effects of the soluble molecules CHIR99021, LDN193189, and FGF-2 in addition to MYOD on the differentiation process in all three human-derived MSC types, we quantified the expression of several marker characteristic of key stages of myogenesis at 3, 7, and 14 days after treatment (Fig. 1).

### Transdifferentiated cells exhibit high transduction efficiency and advanced myogenic phenotype

**Fig. 2:**
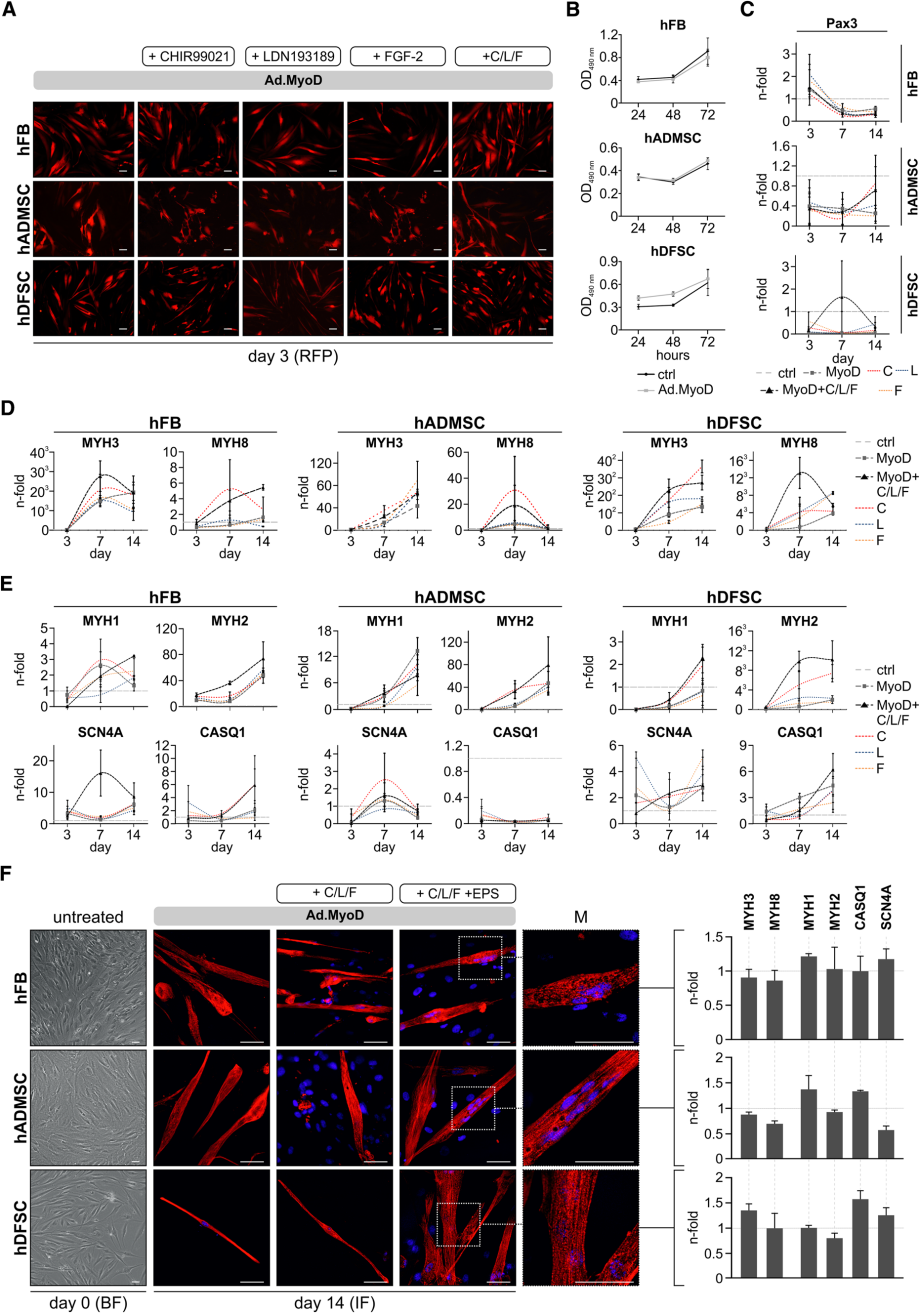
Multifactorial-based reprogramming induces muscle-specific marker gene expression and myogenic transdifferentiation in human-derived stem cells. **A** RFP expression of Ad.MyoD transduced primary cells at day 3 alone or with addition of single soluble factors and the combination thereof (complete). Scale bar: 100 µm **B** Cell viability of the primary cells after Ad.MyoD transduction. **C**–**E** Quantification of transcipt levels in hFB, hDFSC, and hADMSC of indicated differentiation markers at 3, 7 and 14 days representing the MSC **C**, primary **D**, and secondary **E** developmental stage (*n* = 3). Diagrams show the fold change of cells with Ad.MyoD alone (gray dashed line) or after addition of single (C red; L blue; F orange dotted lines) and all soluble factors (black dashed line) compared to untransduced cells (set as 1, dotted horizontal line). **F** Left: untreated cells at day 0. Scale bar: 100 µm; Center: Representative immunofluorescence images of α-actinin (red) and nuclei (blue) showing myofiber maturation in cell types treated with Ad.MyoD alone, in combination with soluble factors (C/L/F) and electrical pulse stimulation (+ EPS) at day 14. Scale bar: 50 µm; M: Magnification of adjacent immunofluorescence pictures. Scale bar: 50 µm; Right: Quantification of transcipt levels of indicated differentiation markers in stimulated cells 14 days post Ad.MyoD transduction (*n* = 3). Fold change is calculated to unstimulated cells (set as 1, dotted line). Expression data are represented as mean ± SD. hDFSC, human-derived dental follicular stem cells; hADMSC, human-derived adipose mesenchymal stem cells; hFBs, human-derived fibroblasts; C CHIR99021; L LDN193189, F FGF-2, EPS electrical pulse stimulation

First, transduction efficiency and transgene expression quality were determined in Ad.MyoD-infected mesenchymal stem cells isolated from fat (adipose mesenchymal stem cells, hADMSC) and dental tissues (dental follicle stem cells, hDFSC) of donors and in primary skin fibroblast (hFB) at different multiplicities of infection (MOIs). After 72 h, red fluorescence staining confirmed complete transduction of all cell types using Ad.MyoD at MOI 100, with no decrease in cell viability (Fig. 2A, B). The clear fluorescent signal, which was still detectable at the same level 14 days after adenoviral infection (data not shown), indicated strong and sustained MYOD expression in hADMSC and hDFSC, comparable to hFB that served as positive control. Addition of CHIR99021, LDN193189, and FGF-2 alone or in combination had no effect on MYOD expression. This demonstrates that MYOD is expressed over the required time period, and that AdV serotype 5 is a reliable gene delivery tool for efficient transduction of adipose and dental stem cells.

To examine the developmental course of cell differentiation induced by multifactor treatment, we quantified the marker genes shown in Fig. 1 compared to untreated cells at 3, 7, and 14 days. As shown in Fig. 2C, we observed a MYOD-dependent downregulation of *PAX3* after day 3 and concomitant increase of *MYH3* and *MYH8,* as markers of primary myogenesis, at day 7 in all three cell types (Fig. 2D). In addition, *MYH1* and *MYH2* transcript levels were increased 7 days after Ad.MyoD transduction and reached a maximum at day 14 of the reprogramming protocol. However, we also found cell type-dependent differences in marker gene expression. In particular, MYOD-expressing hADMSC displayed a significant reduction of *SCN4A* and completely decreased levels of C*ASQ1*. In contrast, hFBs and hDFSC showed a moderate but expected increase of both markers by day 14 (Fig. 2E). Whereas *MYH1* initially decreased in hDFSC compared with the uninduced control at day 3, it was subsequently re-expressed until day 14. Of note, only in hDFSC addition of CHIR99021, LDN193189, and FGF-2 resulted in a clear upregulation of all *MYHC* isoforms after one week (Fig. 2D, E). Together, expression analyses of marker genes at consecutive time points indicate directional muscle-specific development of reprogrammed MSCs and fibroblasts. Our data reveal that MYOD is essential for differentiation induction in these cells and acts as a major driver of early myogenesis. The beneficial effect of the added compounds is particularly evident in hDFSCs for the myosin heavy chain isoforms, demonstrating an advanced onset of myogenic phenotype required for myotube formation.

### Electrophysiological conditioning promotes maturation and purity of functional myotubes

To foster maturation and functionality of reprogrammed cells, we applied electrical pulse stimulation (EPS) on cultured myoblast-like cells (day 7, Fig. 1) to create a natural in vivo environment that simulates potential locomotion stimulation. Therefore, we monitored the morphology and the amount of skeletal muscle protein α-actinin by immunofluorescence (Fig. 2F). Interestingly, all cell populations showed maximal intensities for α-actinin staining and the typical elongated morphology of myotubes 14 days after ectopic MYOD expression alone, in combination with soluble factors, or electrical stimulation. Most notably, we found an increase in multinucleated cells and a more complex structural organization of the skeletal muscle cell apparatus in all EPS-treated cell types. To further assess the effect of electrophysiological conditioning on maturation, we also quantified the specific myosin and ion channel markers of EPS-treated versus unstimulated cells at day 14. No significant changes at the expression level were seen in any of the reprogrammed cell types as demonstrated in Fig. 2F (right panels). These data suggest that application of EPS can enhance myoblast maturation into functional skeletal muscle cells by promoting improved structural and morphological characteristics.

### Transcriptome-based assessment of multifactorial reprogrammed cells using an in silico reconstructed gene regulatory network underlying skeletal muscle phenotype and function

**Fig. 3:**
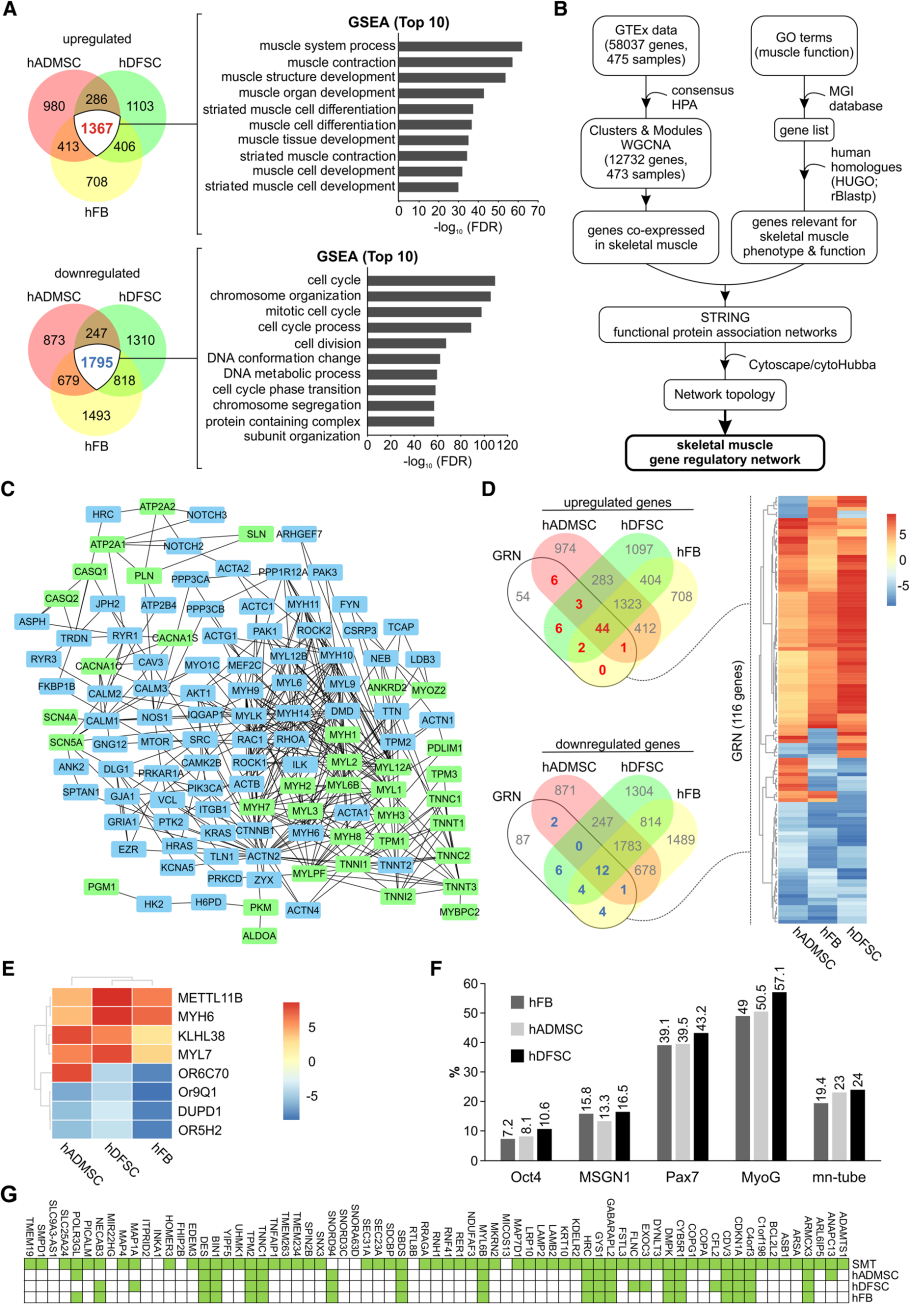
Integrative computational pipeline for the estimation of myodifferentiation based on transcriptomes of multifactorial reprogrammed cells. **A** Comparison among the transcriptomes of hFB-, hADMSC- and hDFSC-derived SMCs. Overlap of the differentially up- (top, left) and downregulated (bottom, left) transcripts as compared to the corresponding uninduced controls. GSEA of commonly up- (top, right) and downregulated (bottom, right) transcripts in the three cell types for GO biological processes. **B** Workflow for in silico reconstruction of a core GRN underlying skeletal muscle phenotype and function. Skeletal muscle transcriptomics data were retieved from publicaly available databases and subjected to gene co-expression and network analysis. For a detailed description see “Material and Methods”. WGCNA, weighted gene co-expression network analysis; HPA, Human Protien Atlas;. GTex, Genotype-Tissue Expression project; GO, gene ontology; MGI, Mouse Genome Informatics database; HUGO, human genome organization; rBLASTp, reciprocal BLASTp; STRING, Search Tool for the Retrieval of Interacting Genes/Proteins **C** The core GRN, entailing 116 hubs coexpressed in normal skeletal muscle and associated with GO terms relevant for skeletal muscle phenotype and function. The green-highlighted nodes represent established markers of muscle fiber type I and II. **D** Left: Venn diagram depicting the differentially up- (upper panel) and downregulated (lower panel) genes from each transdifferentiated cell types in comparison to the 116 genes of the core GRN. Right: corresponding heatmap of the transcriptomes of SMCs produced from hFBs, hADMSCs, and hDFSCs for the 116 genes. **E** Heatmap of the transcriptomes of the transdifferentiated SMCs versus a muscle-restricted expression signature generated from the RNA consensus tissue gene data of the Human Protein Atlas. **F** Percentages of stage-specific genes contained among the differentially upregulated transcripts of the MSCs transdifferentiated by our multifactorial protocol. The stage-specific genes were identified using a recently published stepwise transcriptional blueprint of myogenic specification (Material and Methods). **G** Upregulations of factors crucial for the maintenance of the skeletal muscle phenotype in the transcriptomes of transdifferentiated SMCs. The markers of maintenance of skeletal muscle phenotypes were identified from the stepwise transcriptional blueprint of myogenic specification, as the transcripts with gradually increasing expression toward fully differentiated stages. The transcriptome of the skeletal muscle tissue from the RNA consensus tissue gene data from the Human Protein Atlas was used as a SMT control. GRN, gene regulatory network; SMC, skeletal muscle cells; SMT, skeletal muscle tissue

Induction of the different cell types toward the myo-differentiation lineage is essentially associated with extensive transcriptional reprogramming [5]. Hence, we wondered to which degree the gene expression profiles of the differentiated cells mimic those of normal, functional skeletal muscle and whether any of the initial cell types provides superior reprogramming efficiency. We performed high-throughput analyses on differentiated cells produced either from hADMSCs, hDFSCs, or hFBs. Subsequent comparison of differentially expressed genes (DEG) from the transcriptomes revealed 1367 commonly up- and 1795 commonly downregulated genes in all three cell type-derived muscle cells (Fig. 3A left and Table S7). Gene Set Enrichment Analysis (GSEA) of the commonly upregulated transcripts are strongly associated with myogenic processes, such as muscle contraction, muscle structure development, striated muscle and muscle tissue differentiation and development. In contrast, the commonly downregulated genes were involved in cell cycle processes, chromosome segregation and organization, cell division, and mitotic cell cycle (Fig. 3A right and Table S7), thus corroborating the notion that the received cell populations represent skeletal muscle cells.

For a more detailed understanding of the transcriptional changes in the reprogrammed cells, we set up a bioinformatics workflow to reconstruct a core GRN representative of mature and functional skeletal muscle (Fig. 3B). We used this GRN as a comparator and postulated that the more of the network hubs are expressed in a bioengineered tissue, the more similar this tissue is to a mature and functional skeletal muscle. First, we extended our previously described methodologies [30–32] on publicly available data on mature skeletal muscle to ultimately reconstruct the reference GRN, based on gene co-expression, functionality and network topology criteria (detailed description in “Material and Methods” and Table S3, S4) and created a core GRN of 116 hubs that are co-expressed in normal skeletal muscle and are nodal for skeletal muscle function (Fig. 3C and Supplemental Fig. S1). Then, we compared the reference GRN which we subsequently used to evaluate the obtained transcriptomes to estimate the quality of the differentiated muscle cells. We found that multifactorial reprogramming of hFBs, hADMSCs, and hDFSCs led to an upregulation of 47, 54, and 55 hubs of the reference GRN, respectively. DEGs across all three cell types overlapped to a similar extent with the control list, with 36.21% (42/116) significantly upregulated (Fig. 3D, upper panel; Table S5), and 10.35% (12/116) significantly downregulated (Fig. 3 D, lower panel; Table S5) in each transdifferentiated population.

Of particular interest are the downregulated GRN components in the produced muscle cells (Fig. 3D, blue-colored genes on the heatmap; Table S65). Their loss could lead to significant network perturbations, which in turn would negatively affect the formation of specific network modules associated with mature skeletal muscle function, resulting in incompletely differentiated cells. Interestingly, regulators of glucose uptake in skeletal muscle, such as *MYO1C* [33] and *ROCK1* (Rho-kinase 1) [34], were also found among the downregulated hubs, suggesting limitations in the recapitulation of skeletal muscle metabolism. Evaluation of transcriptomes from transdifferentiated muscle cells with our established GRN allows assessment of the efficiency of the applied protocol and helps to identify missing GRN hubs. Further elucidation of deregulated GRN components may enable identification of new regulatory factors for protocol optimization and cell type-specific improvement of myogenic differentiation.

### Evaluation of the protocol effectiveness based on muscle-restricted gene signatures

To further evaluate the effectiveness of the protocol, we additionally checked whether the transcriptomes of reprogrammed cells include mRNAs that are expressed exclusively in muscle cells, on the basis that expression of muscle-restricted genes is indicative for adequate muscle differentiation. To identify muscle-restricted genes, we used the RNA consensus tissue gene data from the Human Protein Atlas, which contains normalized transcript levels per gene for 62 tissues, and compared the expression of genes in muscle tissues versus all other tissues. Overall, we generated a muscle-restricted signature comprising 8 genes, namely *DUPD1*, *KLHL38*, *METTL11B*, *MYH6*, *MYL7*, *OR5H2*, *OR6C70* and *OR9Q1*. By juxtaposing this signature with the transcriptomes of hFBs, hADMSCs, and hDFSCs-derived SMCs, we found comparable overlaps for all of them, whereby hFBs/hDFSCs express four and hADMSCs five of these gene markers (Fig. 3E).

### Estimation of the maturity of reprogrammed muscle cells using a stepwise transcriptional blueprint of myogenic specification

Expression of exogenous myogenic TFs in pluripotent stem cells generates a mixture of subpopulations from early, mid- and late myo-differentiation stages. To assess this heterogeneity in myogenic cell cultures produced by our multifactor combination, we extrapolated a recently-identified, stepwise transcriptional blueprint of myogenic specification [24] to the differentiated SMC transcriptomes. In detail, Choi et al. characterized the transcriptional landscape of distinct human myogenic stages using well-established marker genes of human myogenesis, in particular *OCT4* and *NANOG* (pluripotent stage), *MSGN1* and *TBX6* (presomite stage), *PAX7* (putative myogenic stem/progenitor cell stage), and *MYOD1* and *MYOG* (myoblast cells before myotube formation). Following an in vitro myogenic specification protocol directing human PSCs into skeletal muscle cells, they isolated cells from distinct stages, including OCT4::EGFP + embryonic stem cells, MSGN1::EGFP + presomite cells, PAX7::EGFP + putative skeletal muscle stem/precursor cells, MYOG::EGFP + myoblast cells, and multinucleated myotubes. By subjecting these subpopulations to single-cell RNA-seq, they defined stage-specific transcriptional dynamics of human myogenesis from undifferentiated PSCs to differentiated myotubes [24].

We hypothesized that within the abovementioned transcriptional blueprint, the genes which start being expressed from each of the consecutive stages and retain their expression until the fully differentiated myotubes might be strongly indicative for cells existing in corresponding stages. Hence, we meta-analyzed the RNA-seq data from each stage of myo-differentiation produced by Choi et al., and screened particularly for genes that are persistently expressed from each of these stages and on (Table S6). These were overlapped with significantly upregulated transcripts from our reprogrammed muscle cells, and their percentage relative to the number of genes specific to each myo-differentiation stage was estimated. In total, of the 5204 genes whose expression starts from each distinct differentiation point, 4252 are expressed at the pluripotent stage (OCT4-TUB), 158 at the presomite stage (MSGN1-TUB), 266 at the myogenic cell stage (PAX7-TUB), 198 at the myogenic stage (MYOG-TUB), while 330 genes are myotube-specific (TUB). We found a high degree of congruence in regard to the cellular heterogeneity of transdifferentiated SMCs from all three cell sources (Fig. 3F), with particular enrichment of the subpopulations in the late myogenic and myotube-specific stages. Notably, hDFSCs produce slightly enhanced late-stage subpopulations, expressing 57.07% and 23.94% genes that characterize the myoblast and myotube stages.

Among the 5204 genes, we noticed a particular subset of 71 genes with gradually increasing expression at different stages of myo-differentiation. The majority (54 genes) begin their expression at the pluripotent stage, while 17 appear at the presomite stage. The tendency for increasing expression during the progress of myo-differentiation indicates that this gene subset may be particularly important for the maintenance of the skeletal muscle cell phenotype. Their significance is underscored by the fact that 86% (61/71) of these genes are expressed in the transcriptome of normal skeletal muscle tissue (SMT), based on HPA-derived RNA data (Fig. 3G). Overlap of these markers with the transcriptomes of transdifferentiated SMCs showed that several of them are induced by the applied protocol, whereas hDFSC-derived cells express slightly higher number of markers (Fig. 3G). In summary, this approach (a) demonstrates that our established protocol activates several stage-specific factors that are critical for commitment to myogenic cell fate and maintaining SMC phenotype; (b) deciphered a slightly higher potential of hDFSCs as a cell source for SMTE compared with hADMSCs and hFBs, and (c) identified factors that are not expressed in the produced cells, but whose reciprocal addition in a subsequent cycle of the multifactorial protocol could potentially enhance their muscle cell phenotype.

### Transdifferentiated SMCs exhibit specific electrophysiological characteristics of mature myotubes

**Fig. 4:**
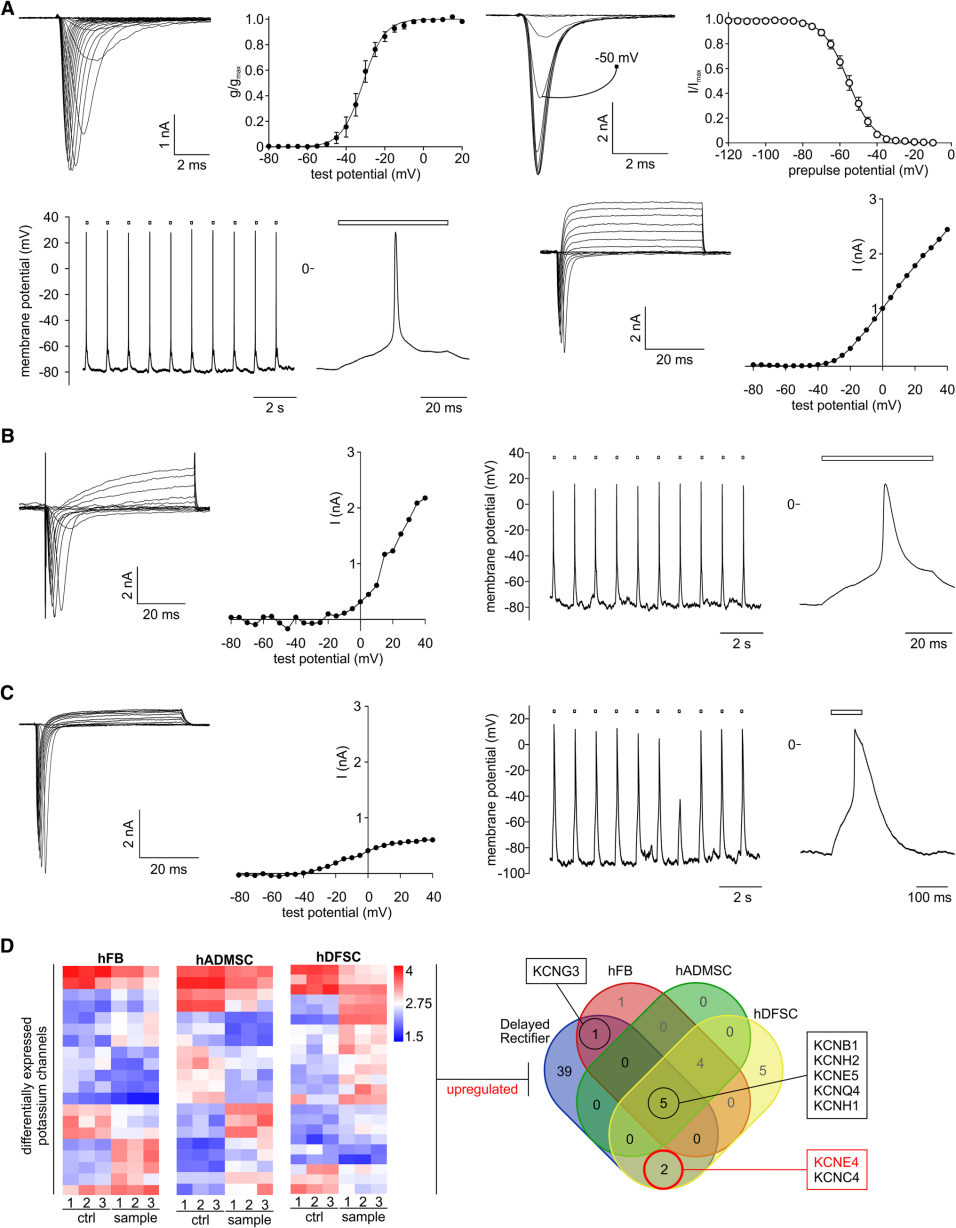
Electrophysiological properties of reprogrammed and transdifferentiated cells. **A** Voltage gated Na^+^ currents, K^+^ currents and action potentials (APs) recorded from hFB-derived myotubes. Upper row, from left to right: family of traces of Na^+^ inward currents induced by square voltage pulses (10 ms) to between −80 mV and + 20 mV. The electrode was filled with an internal solution containing CsCl as main electrolytes. Second: voltage dependence of activation of the Na^+^ currents. Data points were evaluated from current traces as shown most left and a sigmoid curve fitted to the data points. Data from *n* = 7 independent cells; symbols represent means ± SEM. Third plot: Na^+^ inward currents, every second trace plotted, induced by square voltage pulses to −10 mV going from varying pre-pulse potentials. The Na^+^ current transient corresponding to a pre-pulse potential of −50 mV is indicated. Most right: voltage dependence of inactivation of Na^+^ currents derived from the aforementioned recordings. A Boltzmann curve was fitted to the data points showing means ± SEM for *n* = 10 cells. Second row: electrodes were filled with KCl as main electrolytes to record series of APs from a myotube in response to stimulation with depolarizing currents for 100 ms (markers above). Second plot: single AP plotted on a broader time scale. Third plot: family of inward and outward currents elicited by square voltage pulses going from −80 mV to + 40 mV for 50 ms. Na^+^ inward currents showing fast inactivation are followed by late non-inactivating K^+^ outward currents. Current/voltage curve of the K^+^ outward currents calculated from the last 20 ms of the current traces. **B**, **C** Similar sets of data as shown in **A** for hADMSC-derived **B** and hDFSCs-derived myotubes **C**. From left to right: family of Na^+^ inward currents showing fast inactivation followed by late, non-inactivating K^+^ outward currents. Second plots: current/voltage curves of the K^+^ outward currents calculated from the last 20 ms of the current traces. Third plots: series of APs recorded from myotubes in response to depolarizing currents of 100-ms duration (markers above) and single APs plotted on a broader time scale (**B**, **C** most right). **D** Left: Heatmaps of K^+^ channels show similar expression pattern of differentially expressed genes in all three cell types. Right: Comparison of GO term classified delayed rectifier genes with overexpressed K^+^ channels of reprogrammed myotubes

For validation of the bioinformatics analyses and the quality of cells differentiated into functional myotubes/myofibers, we recorded action potentials as well as sodium and potassium currents in single myotubes (Fig. 4A–C). When hFB-derived myotubes were stimulated with depolarizing voltage pulses in the whole-cell configuration, they responded with transient inward currents. Kinetics of the currents was typical for voltage gated Na^+^ channels and in agreement with the functional expression of Na_v_1.4 channels (Fig. 4A, upper row, most left). The voltage dependence of activation of the Na^+^ currents, derived from such recordings, showed an average *V*_1/2_ of −31.2 mV (Fig. 4 A, upper row, 2nd plot). Voltage dependence of steady-state inactivation of Na^+^ currents was tested by applying variable pre-pulses and constant test pulses to −10 mV. Amplitudes of fast Na^+^ inward currents typically declined with increasingly depolarizing pre-pulses without changes in the kinetics of the transients (Fig. 4A, upper row, 3rd plot). *V*_1/2_ of steady-state inactivation yielded on average −55.7 mV (Fig. 4A, upper row, most right). When electrodes were filled with the K^+^ containing internal solution, delayed K^+^ outward currents could be elicited (Fig. 4A, 2nd row, two most right plots). The currents did not inactivate on the timescale of 50 ms and showed current/voltage relations that were typical for voltage gated K^+^ channels (Fig. 4A, 2nd row, most right). After switching the amplifier to the current clamp mode, action potentials could be induced in the same cells. APs were quick, occurring within few ms and showed regularly an overshoot to about + 20 mV (Fig. 4A, lower row, two most left plots). The required depolarizing current pulses were very variable, depending on cell size and membrane potential and ranged between 10 and 100 pA. Induction of APs was very reproducible at 1 Hz (Fig. 4A, most left). No spontaneous APs were observed. Similar results as shown for the hFB-derived myotubes were obtained with the reprogrammed and differentiated hADMSC (Fig. 4B). Myotubes derived from hDFSC were likewise electrically excitable as they showed APs and large Na^+^ inward current (Fig. 4C). However, K^+^ currents were markedly smaller in the hDFSC myotubes compared with those of hFB and hADMSC. In one series of experiments only two out of 10 cells showed significant K^+^ currents. This observation is consistent with the finding of long-lasting APs with very long (100 ms) repolarization phases (Fig. 4C, most left).

To understand the deficiency on K^+^-dependent repolarization, we reanalyzed our array data for DEGs that are related to a certain class of delayed rectifiers. Interestingly, hDFSCs had the highest amount of differentially expressed potassium channels compared with hFBs or hADMSCs (Fig. 4D left; Table S8). Comparing these gene with a GO term-based list of 47 candidates associated with delayed rectifier function, we identified *KCNE4* and *KCNC4* as uniquely upregulated in hDFSCs (Fig. 4D right). Especially, KCNE4 functions as an inhibitory subunit with other K^+^ channel proteins [35, 36], which might relate to the low abundance of K^+^ currents and long repolarization.

## Discussion

MYOD-based reprogramming of PSCs is a well-known strategy of SMTE; however, current protocols face drawbacks, such as low transduction efficacies, insufficient muscle development, and production of mixed population of myogenic cells [5, 37]. Herein, we developed a multifactorial direct reprogramming approach to efficiently produce mature and physiologically functional myotubes from different human-derived somatic stem cells. According to this novel protocol, a baseline Ad.MyoD-induced differentiation process is optimized through time-bound addition of the small molecules CHIR99021 and LDN193189, and FGF-2, which enhance key pathways of embryonic muscle formation, and followed by an additional step of electrical pulse stimulation (EPS) for full maturation of reprogrammed cells. We combined the experimental protocol with an in silico, omics-based workflow, whereby on one hand, we reconstructed a core network, which is essential for skeletal muscle functionality and on the other hand identified stage-specific factors that are crucial for the maintenance of the skeletal muscle phenotype, and juxtaposed them with the transcriptomes of transdifferentiated cells. This systematic approach, complemented by electrophysiology assays, enabled us not only to comprehensively evaluate the quality of produced muscle cells in terms of maturation, functionality and cellular heterogeneity, but, at the same time, to also predict key nodes that are still absent from the cells. These findings allow for dynamic and stepwise improvement of the protocol, since missing key factors can be added subsequently, the transdifferentiated cells can then be re-evaluated for quality, and the remaining missing modulators can be predicted again. Using iterative cycles, we can repeat the workflow until reaching cell phenotypes with enhanced muscle cell functionality.

In this study, as starting cellular source, we used for the first time hDFSCs and compared them to hADMSCs and most commonly used dermal fibroblasts. All three cell types have the advantages of relatively easy accessibility, low immunogenicity, and long-term proliferative activity without losing multipotency, and are suitable for autologous transplantation [38, 39]. Dental stem cells are MSC-like populations with self-renewal capacity that can be isolated from dental pulp, exfoliated deciduous teeth, apical papilla, and dental follicle. Their regenerative attitude renders them appealing sources for tissue engineering [7]. We now showed that stem cells from the dental follicle are non-inferior to fibroblasts and adipose tissue cells in terms of their reprogramming to myocytes, posing as an attractive additional or alternative multipotent cell type for SMTE. Furthermore, the beneficial effects of our multifactorial protocol are underlined by the high efficiency in myogenic differentiation in all cell types tested. Especially, hDFSCs expressed a higher number of genes important for the maturation and maintenance of the skeletal muscle phenotype upon application.

Cells produced via SMTE in an autologous manner could theoretically constitute suitable grafts for patient-specific muscle repair and/or functionality improvement. Nevertheless, it should be kept in mind that skeletal muscle tissue is highly vascularized and innervated, embedded in components of the metabolic and regulatory machinery that support efficient energy production and cellular homeostasis. Muscle health and associated motor activity depend on precisely coordinated activity between these components [4]. As such, integration of ex vivo muscle tissue may still be suboptimal to achieve successful muscle repair, because these cells may be naïve to mingle in this inherently complex and highly structured tissue microenvironment. One approach to bypass this obstacle would be the electrophysiological conditioning of myocytes to the muscle tissue environment through the prior provision of EPS as a means to mimic the natural bioelectrical parameters of the in vivo milieu, such as motion stimulation or activation of motor neurons [40, 41]. In fact, EPS promotes myofiber differentiation through cytoskeletal rearrangements and establishment of a metabolic, more oxidative phenotype [41]. Indeed, when we introduced EPS on cultured myoblast-like cells, we observed an increase of multinucleated cells and a more intricate structural organization of the skeletal muscle cell apparatus in all three cell types. An additional perspective to prepare the engineered myocytes for in vivo integration into skeletal muscle environment would be to further co-culture the reprogrammed muscle cells with relevant cellular components, such as endothelial or neuronal cells. In support of this appealing scenario, a vascularization strategy using co-culture with endothelial cells (ECs) and fibroblasts improved the survival of the bioengineered skeletal muscle tissues [42]. In an analogous manner, when motor neurons derived from induced PSCs are cocultured with enhanced-maturity muscle tissue, they form neuro-muscular junctions with increasing co-localization of pre- and postsynaptic markers, as well as increased frequency and magnitude of synaptic activity. Vice versa, the co-cultured muscle tissues also demonstrated increased expression of genes related to sarcomere maturation and innervation over time [43]. On a similar note, Kim and colleagues used 3D bioprinting to generate human skeletal muscle constructs able to form multi-layered bundles with aligned myofibers that mimic the complex 3D structural organization of skeletal muscle tissue. They further showed that neural cell integration into this construct accelerated functional muscle regeneration via improvement of myofiber formation, long-term survival, and neuro-muscular junction formation. The 3D bioprinted human neural–skeletal muscle constructs showed signs of rapid integration with the host neural network, resulting in accelerated muscle function restoration [42]. Overall, these studies collectively indicate that preconditioning ex vivo produced myocytes in several microenvironmental parameters that are characteristic for skeletal muscles, such as bioelectrical activity, intercellular signaling, and 3D organization, before transferring the implant to the recipient, might hold promise for successful in vivo integration into skeletal muscle.

Given the impact of the muscle tissue environment, limitations of existing therapies in neuromuscular diseases, and the particular suitability of adenoviral vectors for the transfer and expression of reprogramming factors into dental and adipose-derived MSC as well as in fibroblasts, direct in vivo cell reprogramming strategies may also be considered. For this purpose, surface-modified Ad vectors are guided to specific receptors on target cells, blocking binding via their natural receptors on other cells in which potential therapy genes are not to be expressed. Using our Smart-AdV technology [44], we aim to express MYOD together with shRNAs for the small molecule inhibitors that have been identified as optimal factors in our protocol, in targeted AdV. A relevant example of the prospects for success of such an approach in complex living systems, in which differentiating cells can respond to environmental stimuli, adapt, and maintain homeostasis is the direct reprogramming of myofibroblasts into induced hepatocytes previously performed in vivo. In this preclinical study, we used the neurotrophin receptor-derived peptide p75NTR as S11-NGFp fusion protein as linker of AdV expressing four transcription factors [8]. The results showed that liver myofibroblasts were selectively transduced in situ and administration of the specific vector did not affect liver function in healthy mice.

Last but not least, our study underscores a need for nexus of bioinformatics and tissue engineering, which could accelerate translation to clinical therapeutic solutions. Many SMTE approaches are traditionally based on meticulous laboratory work, whereby reprogramming factors or combinations thereof are tested in a trial-and-error manner for their ability to recapitulate the hierarchical networks of myo-differentiation and to hopefully produce autologous tissue grafts suitable for implanting on patients. Notably, advanced computational algorithms that optimize for time, cost and accuracy measures across broad domains of biological data science, as well as open-access benchmarking data are already in place and hold a potential to guide these SMTE protocols. Such tools and sources are anticipated to be increasingly adapted in several research areas, from complex conditions, for example cardiovascular diseases [45] to regenerative medicine. Additionally, current advancements in biomaterials and 3D culture platforms, such as 3D bioprinted constructs [42], organoids, and organ-on-a-chip techniques [46, 47], create unprecedented opportunities for the production of tissue grafts with an enhanced tendency to be integrated to patient skeletal muscles. Systems medicine initiatives that combine these state-of-the art bioengineering protocols with high-throughput data and computational pipelines could lead to the birth of a novel field at the multidisciplinary interface of bioinformatics and SMTE, which we refer to as ‘myoinformatics’, as a necessary complement of SMTE.

## Supplementary Information

Below is the link to the electronic supplementary material.

Supplementary file1 (PDF 1559 KB)

Supplementary file2 (XLSX 114 KB)

Supplementary file3 (XLSX 85 KB)

Supplementary file4 (XLSX 18 KB)

Supplementary file5 (XLSX 364 KB)

Supplementary file6 (XLSX 247 KB)

Supplementary file6 (XLSX 22 KB)

## Data Availability

Data and material are available upon request.
