# Supplementary material for: Novel integrated workflow allows production and in-depth quality assessment of multifactorial reprogrammed skeletal muscle cells from human stem cells
Source: Cell Mol Life Sci. 2022 Apr 9;79(5):229. doi: 10.1007/s00018-022-04264-8 (PMC8993739; doi:10.1007/s00018-022-04264-8)
Supplement: Supplementary file 1 — Supplementary file1 (PDF 1559 KB) [file 18_2022_4264_MOESM1_ESM.pdf]

Supplemental Information

Novel integrated workflow promotes production and assessment of multifactorial reprogrammed skeletal muscle cells from human-derived stem cells

Dinis Faustino[1,2], Heinrich Brinkmeier[3], Stella Logotheti[1], Anika Jonitz-Heincke[4], Hande Yilmaz[1,2], Isil Takan[5,6], Kirsten Peters[7], Rainer Bader[4], Hermann Lang[8], Athanasia Pavlopoulou[5,6], Brigitte M. Pützer[1,2,9,*], Alf Spitschak[1,2,9]

[1]Institute of Experimental Gene Therapy and Cancer Research, Rostock University Medical Center, 18057 Rostock, Germany
[2]Department Life, Light & Matter, University of Rostock, 18059 Rostock, Germany
[3]Institute of Pathophysiology, University Medicine Greifswald, 17489 Greifswald, Germany
[4] Biomechanics and Implant Technology Research Laboratory, Department of Orthopedics, Rostock University Medical Centre, 18057 Rostock, Germany
[5]Izmir Biomedicine and Genome Center (IBG), Balcova 35340 Izmir, Turkey.
[6]Izmir International Biomedicine and Genome Institute, Dokuz Eylül University, Balcova 35340 Izmir, Turkey.
[7]Department of Cell Biology, Rostock University Medical Center, 18057 Rostock, Germany
[8]Department of Operative Dentistry and Periodontology, Rostock University Medical Centre, 18057 Rostock, Germany
[9]Co-senior authors
[*]Correspondence: brigitte.puetzer@med.uni-rostock.de

SUPPLEMENTAL FIGURES

Fig. S1

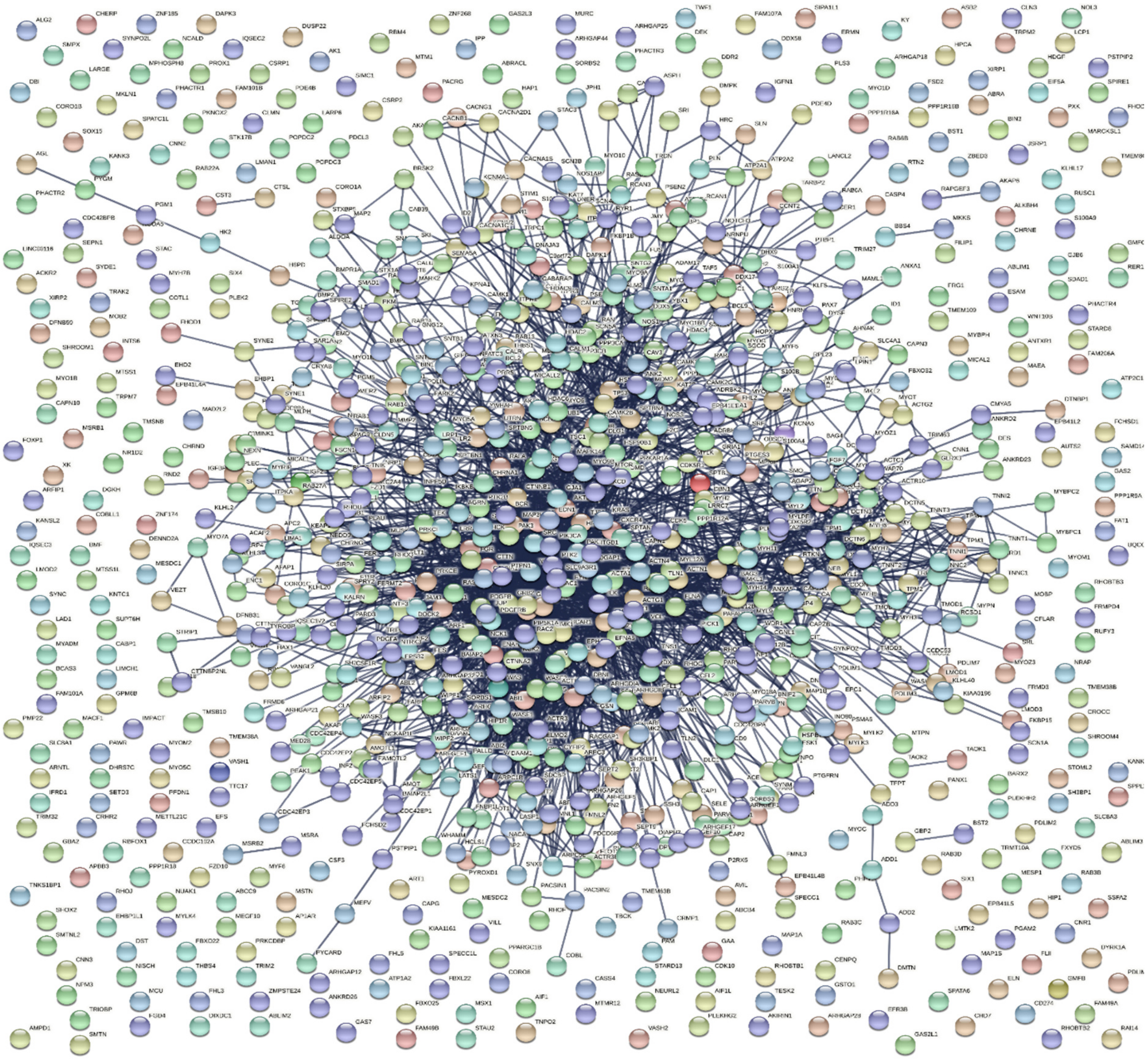

Fig. S1 (related to Fig. 3C) STRING generated reference network used for the establishment of the core regulatory network.

Fig. S2

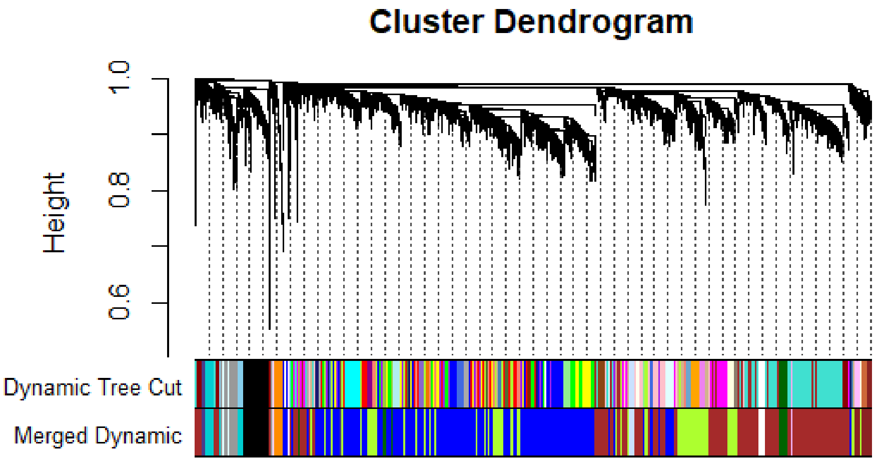

Fig. S2: A dendrogram of a total of 12732 genes and 473 normal samples was used to generate the gene co-expression network of normal skeletal muscle tissue. Gene modules are represented by different colors. The branches in the dendrogram represent modules, and each leaf, which is a short vertical line, corresponds to a gene. A total of 16 gene co-expression modules were determined, labeled as black (516); blue (5023); brown (3422); brown4 (277); dark green (296); dark orange (138); dark slateblue (89); dark turquoise (305); greenyellow (1985); grey60 (255); light cyan1 (75); light steelblue1 (78); plum2 (52); thsitle1 (40); thistle2 (44); white (137). The numbers in the parentheses indicate the number of genes per module. The unclustered genes should have assigned to the grey module. No outlier genes were detected, and all 12732 genes were included for further analysis.

SUPPLEMENTAL TABLE

Table S1 Supplements used for medium composition

	Stock	Final	dilution	amount	Cat. #
RPMI 1640,Glutamax				489 ml	11320-033, Gibco
Zellshield	100 x	1 x	1:100	5 ml	13-0150, MinervaBiolabs
Sodium pyruvate	100 x	1 x	1:100	5 ml	11360070, Thermo Fisher
B27 supplement (with Insulin)	100 x	2 x	1:50	1 ml	17504044 (+ Insulin) Thermo Fisher
Ascorbic Acid	200 mM	200 µM	1:1000	0.5 ml	A8960, Sigma

Table S2 Primers used for RT-qPCR

**Sequence name**	**Sequence**
forh-PAX3	CTCACCTCAGGTAATGGGACT
revh-PAX3	CGTGGTGGTAGGTTCCAGAC
forh-MYH3	GGAGCAGGACAGAAGATAT
revh-MYH3	CCCAGATTGAAACAAAGCA
forh-MYH8	GCACACACCCTCACTTCGTA
revh-MYH8	TCATGTTCCATTGCCCCAGG
forh-MYH1	ATCTAACTGCTGAAAGGTGACC
revh-MYH1	TAAGTACAAAATGGAGTGACAAAG
forh-MYH2	TCTCCAAAGCCAAGGGAAAC
revh-MYH2	TGCGCAGTCAGGTCATTGAT
forh-SCN4A	ACGAAGTGACTTGGAGGCTG
revh-SCN4A	GAACATGCTGAACAGCGCAT
forh-CASQ1	GGAGCACAGGAGATCAACCC
revh-CASQ1	TATCTTGGGCCACAGCCTTG
